# Using the Masseteric Artery to Navigate the Masseteric Nerve in Facial Reanimation Surgery

**DOI:** 10.3390/medicina62010082

**Published:** 2025-12-30

**Authors:** Stefan Rössler, Wolfgang Zemann, Niels Hammer, Veronica Antipova

**Affiliations:** 1 Division of Macroscopic and Clinical Anatomy, Gottfried Schatz Research Center, Medical University of Graz, Auenbruggerplatz 25, A-8036 Graz, Austria; st.roessler@stud.medunigraz.at; 2 Department of Oral and Maxillofacial Surgery, Klagenfurt Am Wörthersee Clinic, Feschnigstraße 11, A-9020 Klagenfurt am Wörthersee, Austria; 3 Department of Oral and Maxillofacial Surgery, Medical University of Graz, Auenbruggerplatz 5, A-8036 Graz, Austria; wolfgang.zemann@medunigraz.at; 4 Department of Orthopedic and Trauma Surgery, University of Leipzig, D-04103 Leipzig, Germany; 5 Division of Biomechatronics, Fraunhofer Institute for Machine Tools and Forming Technology Dresden, D-09126 Dresden, Germany

**Keywords:** clinical anatomy, dissection, face morphology, facial reanimation, maxillofacial surgery, surgical approach, Thiel embalming

## Abstract

*Background and Objectives*: The masseteric artery (Ma) enters the masseter muscle (Mm) together with the masseteric nerve (Mn) via the mandibular notch. Morphological detail on the intramuscular course of the Ma and its relationship to the Mn remains scarce to date. When utilizing the Mn in facial reanimation surgery, a constant relationship between the Ma and Mn could be used for intramuscular orientation when preparing the Mn and for an indirect localization via ultrasound. This study examines the intramuscular course of the Ma and its relationship to the Mn. *Materials and Methods*: Sixty hemicrania obtained from thirty postmortem individuals aged between 54 and 99 years and embalmed using the Thiel methods were examined. *Results:* Four types of Ma were identified according to their endpoint in the Mm. In 5% of cases, no Ma could be identified (Type 0), 48.3% (Type 1) terminated within the upper third, 41.7% (Type 2) in the middle third, and 5% (Type 3) in the lower third. The Ma consistently entered the Mm inferior and in 85% of cases additionally slightly posterior to the Mn. The main trunk of the Ma crossed the Mn in the upper third of the Mm in 31.7% of cases, in the middle third in 23.3%, and in the lower third in 1.7% of cases. Of these, 13.3% had the Ma crossing the Mn. Smaller branches crossed the Mn in 45% of cases. *Conclusions:* If an Ma is present, it may be used for intramuscular orientation and indirect location of the Mn via the mandibular notch. Since the Ma reaches the lower third of the muscle in only a few cases, it is unsuitable for intramuscular orientation to locate the Mn via a distal approach.

## 1. Introduction

The masseteric artery (Ma) forms a branch of the pterygoid segment of the maxillary artery and supplies the masseter muscle (Mm) together with branches of the facial, premasseteric branch of the facial artery, transverse facial, external carotid, maxillary, superficial temporal and deep temporal artery with blood [1]. It lies between the sphenomandibular ligament and the neck of the condylar process. The Ma passes the mandibular notch together with the masseteric nerve (Mn) and a corresponding vein before entering the Mm. Knowledge of the blood supply and innervation of the Mm is of clinical importance for surgical procedures in the field of dysgnathia, reconstruction and reanimation. The proximity of the Ma to the mandibular incisure makes it a potential cause of bleeding during vertical ramus osteotomies [2]. In the field of reconstruction, the possibility of resuscitating the eye using a masseter flap in combination with a fascia graft was already described in an anatomical setting [3]. Further, the Mn is becoming increasingly popular in facial reanimation surgery due to the positive results described in the literature [4,5]. Numerous different anatomical landmarks have been described to be suitable for Mn identification [6,7,8,9,10]. Cotrufo et al. described a so-called “Masseteric Area” measuring 3.36 cm^2^, defined by the inferior margin of the zygomatic arch, a vertical line through the base of the tragus and a horizontal and vertical line through the most distal point of the Mn suitable for coaptation [6]. Collar et al. defined a so-called “Zygomatic Triangle”, defined by the zygomatic arch, a vertical line through the temporomandibular joint and the frontal branch of the facial nerve [7]. Cheng et al. identified the Mn in a 1.5-cm^2^ area defined by the zygomatic arch, condyle, coronoid process and mandibular notch [8]. Borschel et al. stated that the Mn can be found 3 cm anterior to the tragus and 1 cm inferior to the zygomatic arch [9]. This “3:1 rule” was verified in a pediatric population by Mundschenk and colleagues [10]. The landmarks mentioned provide a rough guide to the location of the Mn. However, they are not suitable for intramuscular orientation during dissection. The aim of this study is to examine whether the Ma shows a consistent relationship with the Mn which could be useful for an indirect identification of the nerve via ultrasound and for intramuscular orientation during dissection. Furthermore, an understanding of the vessels surrounding the mandible is necessary to prevent intraoperative bleeding during mandibular resections, osteosynthesis, and osteotomies, and to refine existing surgical techniques.

## 2. Materials and Methods

The present study was approved by the Ethics Committee of the Medical University of Graz, protocol number 35-477 ex 22/23 on 3 October 2023. It was conducted at the Division of Macroscopic and Clinical Anatomy, Gottfried Schatz Research Center, Medical University of Graz (Austria). While alive, all body donors had given their informed consent to the donation of their postmortem tissues for research purposes. All donations were bequeathed to the Division of Macroscopic and Clinical Anatomy of the Medical University of Graz under the approval of the Anatomical Donation Program of the Medical University of Graz and in accordance with the Styrian legislation concerning body donations. Sixty facial halves obtained from 30 postmortem individuals embalmed using the Thiel original and Thiel modified methods with the arterial system injected using a radiopaque red latex mass (70% distilled water and 30% nature latex GIVUL MR (Fa. Helmut Bergk, Frankfurt/Main, Germany)) via the common carotid artery were examined [11,12,13,14]. The age of the eighteen male and twelve female individuals at death ranged from 58 to 94 years. Tissues were only included in this study if they showed no major pathological lesions in the face region, including former surgery, or tumors in the parotideomasseteric region.

### 2.1. Dissection

An incision with a No. 10 scalpel was made starting 2 cm superior to the anterior edge of the helix at the hairline, coursing inferiorly along the hair line to the anterior edge of the helix and continuing to the superior edge of the tragus. At this site, the incision was placed along the posterior edge of the tragus, continuing in an anterior direction at the intertragic incisure following the border of the ear lobe and concha up to the post-auricular hairline. Then, subcutaneous dissection was conducted in an anterior direction to the line of the deep plane approach and lateral border of the orbicularis oculi muscle to create a subcutaneous flap. Dissection started in the superior temporal region for easy access to the subcutaneous plane and continued inferior, where the parotid cutaneous ligament was separated from the cutis. For better flap mobility and visibility of underlying structures, in some cases the incision was extended along the hairline at the anterior and/or posterior end of the marked incision line. Then, a pretragal incision was made down to the parotid fascia, and dissection was conducted in a sub-SMAS plane to visualize the border of the parotid gland, the facial nerve branches, the transverse facial artery and the parotid duct. After identification of the temporal and zygomatic branch of the facial nerve a horizontal incision along the inferior border of the zygomatic arch was made. Then, the zygomatic cutaneous ligament and masseteric cutaneous ligaments were separated from the SMAS-flap and the flap was shifted anteriorly. At this point, the facial nerve branches, transverse facial artery and parotid duct were vaguely visible through the parotideomasseteric fascia. Therefore, the fascia was removed and the aforementioned structures were relocated anteriorly. Then, muscle fibers and aponeurosis of the Mm were removed lateral to the mandibular incisure. After identification of the Ma at the entry into the Mm, the vessel was visualized down to its endpoint.

### 2.2. Morphometric Assessments

**Figure 1:**
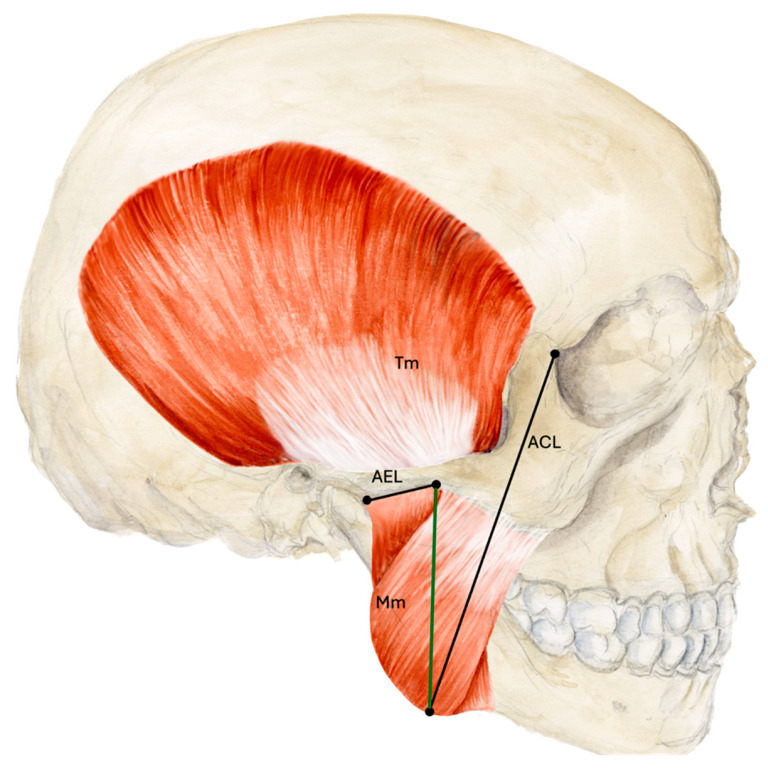
Measurements based on anatomical lines. The thirds of the masseter muscle were measured according to a line (green) connecting the AEL and ACL. Abbreviations: AEL, articular eminence line; ACL, angulus-canthus line; Mm, masseter muscle; Tm, temporalis muscle.

Measurements were taken with a typical surgical ruler, goniometer and caliper in relation to the muscle entry, a line connecting the articular eminence with a point at the inferior border and base of the temporal process of the zygomatic bone (AEL) and a line running from the mandibular angle to the lateral canthus (ACL) (Figure 1). The surgical ruler was used to examine in which third of the Mm the Ma ends according to a vertical line connecting the AEL and ACL and where the Ma crosses the ACL. The goniometer was used to examine the angle at which the proximal Ma runs to the upper edge of the zygomatic arch and the AEL. The caliper was used to determine the diameter of the Ma at the muscle entry and crossing point (CP) with the ACL and the results were rounded to one tenth of a millimeter. The relationship of the Ma to the Mn was assessed directly on the body donors and on the basis of photos taken during and after dissection.

### 2.3. Statistical Analysis

Microsoft Excel version 16.102.2 (25102623, Microsoft Corp., Armonk, NY, USA) was used to collect and analyze the data. Data was collated in tables and processed via Microsoft Excel functions. Data was expressed in means with standard deviations, ranges and percentages.

## 3. Results

### 3.1. Different Types of the Ma

**Figure 2:**
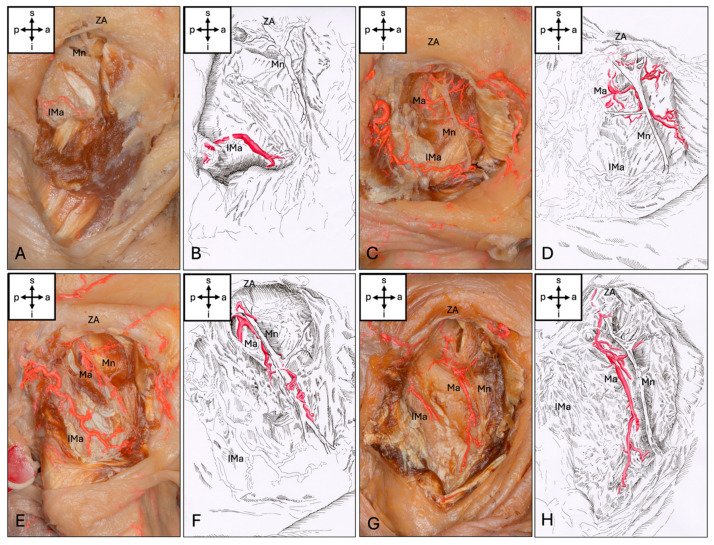
Types of masseteric artery. (**A**,**B**) No artery passes the mandibular incisure (Type 0). (**C**,**D**) The Ma ends in the upper third of the Mm (Type 1). (**E**,**F**) The Ma ends in the middle third of the Mm (Type 2). (**G**,**H**) The Ma ends in the lower third of the Mm (Type 3). Abbreviations: a, anterior; p, posterior; s, superior; i, inferior; Mn, masseteric nerve; Ma, masseteric artery; IMa, inferior masseteric artery; ZA, zygomatic arch.

**Figure 3:**
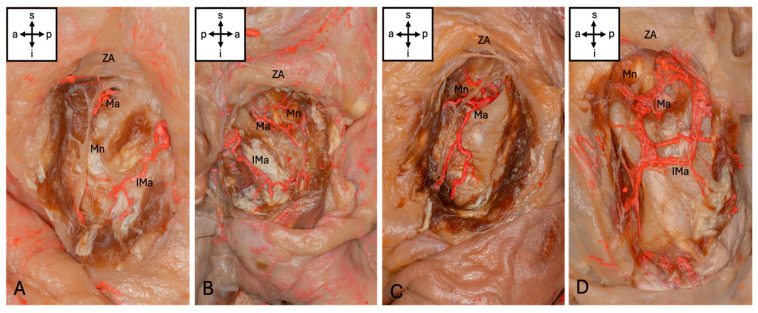
Sub-types of masseteric artery. (**A**) The Ma ends at the level of the mandibular incisure (Type 1a). (**B**) The Ma forms an arcade with an inferior masseteric artery (Type 2b). (**C**) The Ma shows a prominent posterior branch but an inferior masseteric artery is missing (Type 3a). (**D**) The Ma shows an anastomosis with the facial artery and an inferior masseteric artery. Abbreviations: a, anterior; p, posterior; s, superior; i, inferior; Mn, masseteric nerve; Ma, masseteric artery; IMa, inferior masseteric artery; ZA, zygomatic arch.

The Ma could be visualized in 57 (95%) of the hemicrania examined. The Ma terminated intramuscularly in the upper third of the muscle in 29 (48.3%), in the middle in 25 (41.7%) and in the lower third in 3 (5%) cases. In the upper third, 13 (21.7%) terminated at the level of the notch. Anastomoses to other vessels were present in eight (13.3%) cases: six (10%) hemifaces showed anastomoses to an inferior masseteric artery branching from the superficial temporal artery (STA), two (3.3%) hemifaces showed anastomoses to the facial artery, and one (1.6%) showed anastomoses to both vessels mentioned. This inferior masseteric artery was present in 54 cases (90%). Accordingly, four types of artery could be classified (Figure 2). Additionally, four special sub-types could be found (Figure 3).

### 3.2. The Position of the Ma Relative to the Mn

The Ma entered the Mm inferior to the Mn in 100% of cases. Where present, the entry of the Ma into the muscle was slightly posterior to the Mn in 51 cases (85%) and directly inferior to the Mn in 6 cases (10%) (Table 1). The main trunk of the Ma crossed the Mn in the upper third in 19 (31.7%) cases, in the middle third in 14 (23.3%) cases and in the lower third in 1 (1.7%) case, respectively. In 23 cases (38.3%), there was no crossing point of the main branch of the Ma with the Mn. The main trunk of the Ma ran deep to the nerve in 49 (81.6%) cases throughout its intramuscular course, while 8 (13.3%) cases crossed over the Mn. Branches of the Ma crossed over the Mn in 27 (45%) cases.

### 3.3. The Diameter of the Ma and Its Angle to the Zygomatic Arch and AEL

The diameter of the Ma at the muscle entry point averaged 0.8 ± 0.4 mm (0.4–2.1 mm) and 0.8 ± 0.4 mm (0.3–1.9 mm) at the CP with the ACL. The Ma reached the ACL in 17 (28.3%) cases. The Ma ran at an angle of 59.7 ± 22.1° to the zygomatic arch and 66.8 ± 22.1° to the AEL at its proximal part.

## 4. Discussion

### 4.1. Arterial Roadmaps to Nerve Safety

The use of arteries as intraoperative landmarks to identify the nerve location in head and neck surgery has already been described in the literature: Patra et al. found the inferior thyroid artery to be a landmark for protecting the recurring laryngeal nerve during thyroid surgery as the nerve was deep (65%), superficial (30%) or between (5%) the branches of the inferior thyroid artery, respectively [15]. Liu et al. described the posterior auricular artery to be a reliable landmark in an anatomical study for facial nerve identification as it crosses the nerve 5.2 ± 0.2 mm away from the external meatal cartilage [16]. They then tested their results in a clinical setting and were able to locate the facial nerve in 90.3% of parotidectomies via the artery [17]. Only 5.7% of patients experienced postoperative transient facial nerve dysfunction [17].

### 4.2. The Use of the Masseteric Artery to Indirectly Locate the Masseteric Nerve

According to Rajab et al., the distance of the Ma to the antero-superior region of the condylar neck averaged 10.3 ± 4.4 mm (4.1–17.5 mm) and 11.47 ± 4.6 mm (2.8–19.2 mm) to the most inferior point of the articular tubercle and only 3.0 ± 1.2 mm (1.3–5.6 mm) to the most inferior point of the sigmoid notch [2]. Hwang et al. found the Ma 7.8 ± 2.6 mm and the Mn 11.3 ± 2.6 mm above the mandibular notch [18]. Their findings were consistent with the observations presented here, since the main trunk of the Ma in our studies was always inferior and deep in relation to the Mn at the muscle entry, where present. Hwang et al. found the Mn to intersect the Main 69.6% of cases, while we observed a crossing in 56.7% [19]. The constant ratio of Ma to Mn at the level of the incisura makes the artery a useful guide for locating the Mn via the mandibular notch. For access to the Mn via the ACL as described here, the Ma was only suitable for intramuscular orientation to a limited extent, as it only reaches this line in a few cases.

### 4.3. Blood Supply of the Masseter Muscle by the Four Variants of the Masseteric Artery

In the given study, no artery entering the Mm via the mandibular incisura was found in 5% of cases. Using Sihler’s method, Won and colleagues reported the masseteric branch of the maxillary artery to be present in 100% of cases, supplying the deep upper part of the Mm [1]. According to Won et al. the mean diameter of the Ma is 0.7 mm [1]. We measured an average diameter of 0.8 mm at the entry into the muscle. Our study also observed the Ma, where present, to supply the upper third of the Mm in 100% of cases, but additionally supplying the middle (41.7%) and lower third (5%) of the muscle.

### 4.4. Anastomoses of the Masseteric Artery

Won et al. observed that the Mm was supplied by up to seven different arteries, although existing intramuscular anastomoses were not discussed in detail [1]. The given investigations showed anastomoses to other vessels present in eight (13.3%) cases. Fifty-four cases showed an inferior masseteric artery running from the posterior to the anterior edge of the ascending mandibular ramus inferior to the Ma. According to the hypothesis outlined here, this artery was the same as described by Won et al. as the masseteric branch of the transverse facial artery or masseteric branch of the superficial temporal artery [1]. This inferior masseteric artery showed a close positional relationship to the bone in its posterior region and lay significantly deeper than the Ma under an aponeurosis of the Mm. Therefore, its position must be considered during vertical ramus osteotomies, fractures of the mandibular ramus and mandibular resections in order to prevent bleeding.

### 4.5. Approaches for Future Studies

The possibility of resuscitating the eye using a masseter flap in combination with a fascia graft in an anatomical setting has already been described [3]. Expanding our understanding of the blood supply to the Mm could also influence its use in reconstructive surgery in relation to flap elevation. Our macroscopic evidence of the presence of a lower masseteric artery near the bone clearly demonstrates its relevance in the surgical treatment of mandibular fractures. When using a transparotideal approach for mandibular fractures, it is particularly important to avoid damaging the inferior masseteric artery in order to prevent bleeding and damage to the facial nerve caused by cauterization. Since the discovery of this artery in this study was purely coincidental due to its proximity and possible anastomosis to the masseteric artery, further studies would be necessary to establish landmarks that would allow this artery to be spared during surgical procedures.

## 5. Conclusions

To our knowledge, this is the first work to describe the various possible types and courses of the Ma and the possibility of using its constant relationship with the Mn for nerve identification in facial reanimation surgery. If the Mn cannot be identified intraoperatively despite the use of well-described landmarks, this relationship can be used for identification. Based on the findings outlined here, the following recommendations can be made for such situations: after localization of the Ma via blunt dissection or sonography, dissection should be continued superior and anterior to the artery to identify the Mn. Since the masseteric artery reaches the lower third of the muscle only in a few cases, it is not suitable for intramuscular orientation to locate the Mn via a distal approach.

## Figures and Tables

**Table 1:** Information about the masseteric artery.

Types of the masseteric artery	Type 0 (5%) Type 1 (48.3%) Type 2 (41.7%) Type 3 (5%)
Relation to the masseteric nerve at the muscle entry	Directly inferior to nerve (85%) Inferior and posterior to nerve (10%) Masseteric artery not present (5%)
Crossing of the masseteric artery with the masseteric nerve according to muscle third	Upper third (31.7%) Middle third (23.3%) Lower third (1.7%) No crossing (38.3%)
Intramuscular relation of the main trunk of the masseteric artery to the masseteric nerve	Deep to nerve (81.6%) Crossing over the nerve (13.3%)
Diameter	At muscle entry: 0.8 ± 0.4 mm At the ACL: 0.8 ± 0.4 mm
Angle to zygomatic arch	59.7 ± 22.1°
Angle to AEL	66.8 ± 22.1°
Crossing with the ACL	28.3%
Anastomoses to other vessels	To IMa: 10% To facial artery: 3.3%

## Data Availability

Data will be available under reasonable request to corresponding authors.

## Abbreviations

The following abbreviations are used in this manuscript:

ACL	Angle-canthus line
AEL	Articular eminence line
CP	Crossing point
IMa	Inferior masseteric artery
Ma	Masseteric artery
Mm	Masseter muscle
Mn	Masseteric nerve
SMAS	Superficial musculoaponeurotic system
Tm	Temporalis muscle
ZA	Zygomatic arch
